# Supplementary material for: The Detrimental Clinical Associations of Anxiety and Depression with Difficult Asthma Outcomes
Source: J Pers Med. 2022 Apr 26;12(5):686. doi: 10.3390/jpm12050686 (PMC9142921; doi:10.3390/jpm12050686)
Supplement: Supplementary file 1 [file jpm-12-00686-s001.zip › jpm-1658771-supplementary.pdf]

Online Supplementary Material

Table S1. Diagnostic definitions Used in Clinical Practice for Comorbidities.

Comorbidities	Diagnostic Criteria
Rhinitis	Physician clinical diagnosis
Eczema	Physician clinical diagnosis
ABPA	Determined clinically using evidence of relevant serological and radiological information guided by conventional clinical diagnostic criteria
Bronchiectasis	Objective evidence (Radiological Features on any CT imaging of Chest including but not exclusive to HRCT)
COPD	Physician clinical diagnosis
GORD	Physician clinical diagnosis
Dysfunctional Breathing	Physician clinical diagnosis (supported by abnormal Nijmegen score)
Intermittent Laryngeal Obstruction	Physician clinical diagnosis
Sulphite Sensitivity	Physician clinical diagnosis based on compatible history of reaction on exposure and consistent clinical phenotypes
Salicylate Sensitivity	Physician clinical diagnosis based on compatible history of reaction on exposure and consistent clinical phenotypes
Sleep Apnoea	Physician clinical diagnosis (confirmed by sleep study)

ABPA: Allergic Bronchopulmonary Aspergillosis. COPD: Chronic Obstructive Pulmonary Disease. GORD: Gastroesophageal reflux disease.

Table S2. Spirometry, FENO, Eosinophil and Total Ige counts of WATCH patients by clinical diagnoses of psychological comorbidity.

	Anxiety Alone (N = 35, 6.99%)		Depression Alone (N = 51, 10.7%)		Dual Anxiety & Depression (N = 104, 20.8%)		Control (N = 251, 50.1%)	
	Mean (SD)/ Median (IQR)	Missing	Mean (SD)/ Median (IQR)	Missing	Mean (SD)/ Median (IQR)	Missing	Mean (SD)/ Median (IQR)	Missing
FeNO at enrolment, Median (IQR) - ppb	19.0 (24.0)	12	12.7 ** (21.7)	8	18.9 * (25.4)	36	23.5 (35.5)	86
Total IgE levels at enrolment, Median (IQR) - (KU/I)	153 (528)	9	54.8 (120)	14	75.1 (325)	30	67.1 (229)	81
Eosinophil Count at enrolment , Median (IQR) - cells/μL	0.20 (0.20)	6	0.20 (0.20)	8	0.20 (0.30)	18	0.20 (0.20)	53
Pre-BD FEV_1, Mean (SD) - %	76.2 (22.4)	24	54.6 (19.4)**	33	69.2 (27.7)	78	74.9 (22.7)	166
Clinic (Post-BD) FEV_1, Mean (SD) - %	75.2 (37.7)	9	70.1 (20.3)	15	75.1 (21.8)	38	76.2 (23.1)	82
Pre-BD FVC, Mean (SD) - %	110 (56.6)	23	74.6 (20.8)*	33	80.5 (24.8)	78	89.5 (20.0)	166
Clinic (Post-BD) FVC, Mean (SD) - %	94.3 (17.1)	9	84.1 (17.5)*	15	88.8 (16.6)	38	91.5 (19.5)	82
Pre-BD MEF_{25-75}, Median (IQR) - %	48.7 (66.9)	24	23.7 (21.9)**	33	44.5 (60.8)	78	43.6 (46.2)	168
Clinic (Post-BD) MEF_{25-75}, Median (IQR) - %	42.7 (62.7)	9	42.8 (41.1)	15	47.6 (54.1)	38	47.3 (47.3)	82

Significance when compared to the control group: *: $p < 0.050$. **: $p < 0.005$. Continuous variables were expressed as median and interquartile range (IQR) or mean and standard deviation (SD). IgE: Immunoglobulin E. FeNO: Fraction exhaled Nitric Oxide. Continuous variables were analyzed with Mann-Whitney U or independent t-tests where appropriate. Categorical variables were analyzed by Chi-squared tests or Fisher's exact test.

Table S3. Spirometry and FENO of those with a clinical diagnosis of depression by HADS-D stratification.

	HADS-D Normal (0–7) *N* = 79, 54.1%		HADS-D Mild (8–10) *N* = 25, 17.1%		HADS-D Moderate (11–14) *N* = 30, 20.5%		HADS-D Severe (>15) *N* = 12, 8.2%		*p*-Value
	Mean (SD)/ Median (IQR)	Missing	Mean (SD)/ Median (IQR)	Missing	Mean (SD)/ Median (IQR)	Missing	Mean (SD)/ Median (IQR)	Missing	
FeNO at enrolment, Median (IQR) - ppb	19.50 (23.35)	18	16.5 (19.13)	9	17.30 (27.3)	7	8.9 (38.98)	2	0.627
Pre-BD FEV_1 predicted, Mean (SD) - %	74.12 (26.08)	10	70.43 (27.86)	10	59.43 (38.78)	23	78.53 (22.49)	16	0.723
Clinic (Post-BD) FEV_1 predicted, Mean (SD) - %	64.17 (25.07)	29	75.72 (21.75)	2	80.41 (20.57)	13	72.86 (20.11)	4	0.598
Pre-BD FVC predicted, Mean (SD) - %	85.85 (24.39)	10	86.66 (23.98)	10	71.65 (36.56)	23	81.64 (17.97)	16	0.772
Clinic (Post-BD) FVC predicted, Mean (SD) - %	89.02 (18.33)	29	86.42 (13.88)	2	91.27 (19.17)	13	75.99 (17.51)	4	0.197
Pre-BD MEF_{25-75}, Median (IQR) - %	50.58 (57.62)	10	48.02 (52.24)	10	29.72 (58.12)	23	76.84 (114.34)	16	0.574
Clinic (Post-BD) MEF_{25-75}, Median (IQR) - %	40.84 (54.15)	29	62.15 (49.42)	2	44.39 (44.34)	13	49.28 (37.60)	4	0.325

Continuous variables were expressed in median and interquartile range (IQR) or mean and standard deviation (SD) and analyzed with One-way ANOVA or Kruskal-Wallis test + post-hoc analyses. Categorical variables expressed as Percentage (frequency) and analyzed by Chi-squared tests and post-hoc analyses. BD: Bronchodilator. FEV_1: forced expiratory volume in one second. FVC: forced vital capacity. MEF_{25-75}: Mean Mid Forced Expiratory flow rate. FeNO: Fractional exhaled Nitric Oxide.

Table S4. Spirometry and FENO of those with a clinical diagnosis of dual anxiety and depression by HADS-D stratification.

	HADS-D Normal (0–7) *N* = 49, 53.8%		HADS-D Mild (8–10) *N* = 12, 13.2%		HADS-D Moderate (11–14) *N* = 21, 23.1%		HADS-D Severe (>15) *N* = 9, 9.9%		*p*-Value
	Mean (SD)/ Median (IQR)	Missing	Mean (SD)/ Median (IQR)	Missing	Mean (SD)/ Median (IQR)	Missing	Mean (SD)/ Median (IQR)	Missing	
FeNO at enrolment, Median (IQR) - ppb	21.65 (35.18)	13	20.1 (8.95)	6	17.8 (30.95)	5	19 (37.3)	2	0.981
Pre-BD FEV_1 predicted, Mean (SD) - %	72.75 (30.06)	34	70.13 (30.73)	9	71.66 (25.65)	16	57.24 (NA)	8	0.965
Clinic (Post-BD) FEV_1 predicted, Mean (SD) - %	75.03 (23.32)	20	79.54 (20.52)	1	80.89 (19.73)	8	68.00 (21.14)	3	0.624
Pre-BD FVC predicted, Mean (SD) - %	81.53 (27.64)	34	89.93 (13.97)	9	83.30 (24.19)	16	57.98 (NA)	8	0.764
Clinic (Post-BD) FVC predicted, Mean (SD) - %	**91.72** (16.65)	20	91.94 (13.61)	1	92.35 (18.76)	8	*69.18* (69.16)	3	0.020
Pre-BD MEF_{25-75}, Median (IQR) - %	50.58 (72.45)	34	25.46 (NA)	9	34.75 (55.8)	16	53.41 (NA)	8	0.907
Clinic (Post-BD) MEF_{25-75}, Median (IQR) - %	45.84 (66.71)	20	55.92 (65.03)	1	46.99 (45.34)	8	41.22 (33.69)	3	0.666

Continuous variables were expressed in median and interquartile range (IQR) or mean and standard deviation (SD) and analyzed with One-way ANOVA or Kruskal-Wallis test + post-hoc analyses. Categorical variables expressed as Percentage (frequency) and analyzed by Chi-squared tests and post-hoc analyses. For significant differences, highest values indicated in bold and lowest in italics.

BD: Bronchodilator. FEV_1: forced expiratory volume in one second. FVC: forced vital capacity. MEF_{25-75}: Mean Mid Forced Expiratory flow rate. FeNO: Fractional exhaled Nitric Oxide.

Table S5. Asthma outcomes of those with a clinical diagnosis of anxiety by HADS-A stratification.

	**HADS-A Normal (0–7)** *N* =39, 31.7%		**HADS-A Mild (8-10)** *N* =24, 19.5%		**HADS-A Moderate (11–14)** *N* =32, 26%		**HADS-A Severe (>15)** *N* =28, 22.8%		***p*-Value**
	**Mean (SD)/ Median (IQR)**	**Missing**	**Mean (SD)/ Median (IQR)**	**Missing**	**Mean (SD)/ Median (IQR)**	**Missing**	**Mean (SD)/ Median (IQR)**	**Missing**	
Number of OCS courses in the past 12 months, Median (IQR)	1.5 (4)	3	3 (6)	1	4 (4)	1	3 (5.75)	4	0.079
Numbers of days lost from work/education in the past 12 months, Median (IQR)	1 (14.25)	13	9.5 (20)	12	5 (50)	17	14 (90)	17	0.473
Numbers of hospitalizations the past 12 months, Median (IQR)	0 (1)	0	0 (1)	1	0 (2)	0	0 (1)	0	0.783
ACQ6 at enrolment, Median (IQR)	2.5 (2)	2	3 (1.5)	0	3.25 (1.35)	2	3.3 (2)	1	0.006
SGRQ Score (Symptoms) at enrolment, Mean (SD)	60.51 (18.60)	9	73.11 (13.81)	3	72.63 (21.67)	1	79.69 (11.86)	4	0.001
SGRQ Score (Activity) at enrolment, Mean (SD)	64.43 (22.91)	11	83.84 (13.70)	4	78.58 (13.46)	3	92.82 (18.03)	4	0.026
SGRQ Score (Impacts) at enrolment, Mean (SD)	39.78 (17.52)	10	53.72 (17.45)	3	54.65 (17.08)	3	73.36 (18.65)	4	<0.001
SGRQ Score (Total) at enrolment, Mean (SD)	51.02 (16.20)	13	66.43 (13.85)	4	64.65 (13.95)	3	80.43 (14.47)	4	<0.001
FeNO at enrolment, Median (IQR)	21.15 (45.68)	13	20.3 (25.15)	11	21.5 (23.7)	7	14 (15.18)	8	0.225
Pre-BD FEV_1 predicted, Mean (SD) - %	73.93 (24.33)	25	66.06 (26.02)	19	68.15 (40.72)	26	78.41 (19.12)	20	0.836
Clinic (Post-BD) FEV_1 predicted, Mean (SD) - %	77.06 (23.21)	12	73.63 (24.13)	8	71.39 (23.45)	11	78.15 (21.47)	10	0.774
Pre-BD FVC predicted, Mean (SD) - %	100 (54.82)	25	87.23 (20.81)	19	79.95 (38.51)	26	85.09 (16.50)	20	0.728
Clinic (Post-BD) FVC predicted, Mean (SD) - %	94.85 (15.23)	12	91.10 (18.83)	8	84.35 (17.97)	11	90.39 (16.21)	10	0.213
Pre-BD MEF_{25-75}, Median (IQR) - %	49.62 (57.64)	25	25.46 (52.86)	19	40.26 (50.75)	11	50.55 (66.45)	20	0.626
Clinic (Post-BD) MEF_{25-75}, Median (IQR) - %	39.29 (61.17)	12	40.95 (61.74)	8	35.79 (77.44)	26	61.94 (81.73)	10	0.440
	**Percentage (frequency)**	**Missing**	**Percentage (frequency)**	**Missing**	**Percentage (frequency)**	**Missing**	**Percentage (frequency)**	**Missing**	***p*-Value**
ICU admissions for asthma, Ever	20.5% (8)	0	20.8% (5)	0	40.6% (13)	0	35.7% (10)	0	0.185
Intubation for asthma, Ever	12.8% (5)	0	8.3% (2)	0	15.6% (5)	0	14.3% (4)	0	0.873
On mOCS at enrolment	28.6% (10)	4	21.7% (5)	1	35.7% (10)	4	42.9% (12)	0	0.397

Continuous variables were expressed in median and interquartile range (IQR) or mean and standard deviation (SD) and analyzed with One-way ANOVA or Kruskal-Wallis test + post-hoc analyses. Categorical variables expressed as Percentage (frequency) and analyzed by Chi-squared tests and post-hoc analyses. OCS: oral corticosteroids. ACQ6: asthma control questionnaire 6. BD: Bronchodilator. FEV_1: forced expiratory volume in one second. FVC: forced vital capacity. MEF_{25-75}: Mean Mid Forced Expiratory flow rate. FeNO: Fractional exhaled Nitric Oxide. ICU: Intensive care unit.

Table S6. Asthma outcomes of those with a clinical diagnosis of anxiety dual anxiety and depression by HADS-A stratification.

	**HADS-A Normal (0-7) *N* = 26, 28.9%**		**HADS-A Mild (8-10) *N* = 14, 15.6%**		**HADS-A Moderate (11-14) *N* = 28, 31.1%**		**HADS-A Severe (>15) *N* = 22, 24.4%**		***p*-Value**
	**Mean (SD)/ Median (IQR)**	**Missing**	**Mean (SD)/ Median (IQR)**	**Missing**	**Mean (SD)/ Median (IQR)**	**Missing**	**Mean (SD)/ Median (IQR)**	**Missing**	
Number of OCS courses in the past 12 months, Median (IQR)	1.5 (3)	2	3.5 (6.25)	0	3.5 (4.75)	0	3 (7.25)	4	0.299
Numbers of days lost from work/education in the past 12 months, Median (IQR)	2 (13)	10	5 (20)	7	7.5 (53.25)	14	14 (60)	13	0.433
Numbers of hospitalizations the past 12 months, Median (IQR)	0 (1)	0	0 (1)	0	0 (2)	0	0 (1)	0	0.656
ACQ6 at enrolment, Median (IQR)	2.4 (2.1)	0	3 (1.88)	0	3.2 (1.15)	2	3.3 (1.6)	1	0.018
SGRQ Score (Symptoms) at enrolment, Mean (SD)	61.72 (20.84)	2	71.13 (13.85)	1	76.19 (14.87)	4	75.12 (14.88)	3	0.017
SGRQ Score (Activity) at enrolment, Mean (SD)	66.80 (24.71)	2	72.10 (19.70)	1	73.22 (19.09)	6	82.76 (15.98)	4	0.108
SGRQ Score (Impacts) at enrolment, Mean (SD)	37.81 (17.39)	2	51.77 (19.16)	1	53.80 (18.17)	5	59.85 (17.90)	4	0.001
SGRQ Score (Total) at enrolment, Mean (SD)	50.53 (17.21)	1	61.18 (15.38)	1	63.54 (16.22)	6	69.10 (14.12)	4	0.003
FeNO at enrolment, Median (IQR)	22.5 (45.83)	10	20.15 (51)	4	22 (22)	5	12.7 (15.7)	7	0.117
Pre-BD FEV_1 predicted, Mean (SD) - %	74.12 (26.08)	10	70.43 (27.86)	10	59.43 (38.78)	23	78.53 (22.49)	16	0.723
Clinic (Post-BD) FEV_1 predicted, Mean (SD) - %	79.67 (22.15)	17	76.66 (24.71)	4	71.70 (21.33)	10	77.21 (20.72)	8	0.758
Pre-BD FVC predicted, Mean (SD) - %	85.85 (24.39)	10	86.66 (23.98)	10	71.65 (36.56)	23	81.64 (17.97)	16	0.772
Clinic (Post-BD) FVC predicted, Mean (SD) - %	96 (16.20)	17	92.30 (21)	4	83.28 (15.13)	10	87.76 (17.17)	8	0.174
Pre-BD MEF_{25-75}, Median (IQR) - %	50.58 (57.62)	10	48.02 (52.24)	10	29.72 (58.12)	23	76.84 (114.34)	16	0.574
Clinic (Post-BD) MEF_{25-75}, Median (IQR) - %	47.10 (65.21)	17	36.87 (66.14)	4	41.22 (47.50)	10	51.94 (79.83)	8	0.635

	Percentage (frequency)	Missing	Percentage (frequency)	Missing	Percentage (frequency)	Missing	Percentage (frequency)	Missing	p-Value
ICU admissions for asthma, Ever	15.4% (4)	0	14.3% (2)	0	42.9% (12)	0	36.4% (8)	0	0.071
Intubation for asthma, Ever	0% (0)	0	7.1% (1)	0	17.9% (5)	0	13.6% (3)	0	0.154
On mOCS at enrolment	30.4% (7)	3	28.6% (4)	0	37.5% (9)	4	40.9% (9)	0	0.833

Continuous variables were expressed in median and interquartile range (IQR) or mean and standard deviation (SD) and analyzed with One-way ANOVA or Kruskal-Wallis test + post-hoc analyses. Categorical variables expressed as Percentage (frequency) and analyzed by Chi-squared tests and post-hoc analyses. OCS: oral corticosteroids. ACQ6: asthma control questionnaire 6. BD: Bronchodilator. FEV_1: forced expiratory volume in one second. FVC: forced vital capacity. MEF_{25-75}: Mean Mid Forced Expiratory flow rate. FeNO: Fractional exhaled Nitric Oxide. ICU: Intensive care unit.

Table S7. Clinical characteristics of those with Severe psychological comorbidities vs No psychological comorbidities.

	Severe psychological comorbidities N = 36		No psychological comorbidities N = 149		p-Value
	**Mean (SD)/Median (IQR)**	**Missing**	**Mean (SD)/Median (IQR)**	**Missing**	
Age, Median (IQR)- years	52.0 (23.0)	0	59.0 (21.0)	0	0.013
Age of asthma onset, Median (IQR) - years	20.0 (30.5)	3	24.0 (41.0)	8	0.142
Body Mass Index, Median (IQR) – kgm^{-2}	31.2 (12.2)	1	27.9 (7.40)	1	0.005
Number of exacerbations in the past 12 months, Median (IQR)	3.00 (5.00)	5	2.00 (3.00)	12	0.092
Numbers of days lost from work/education in the past 12 months, Median (IQR)	16.0 (48.8)	22	4.00 (14.0)	61	0.026
Numbers of hospitalizations the past 12 months, Median (IQR)	0.00 (1.00)	0	0.00 (0.00)	1	0.720
Duration of asthma, Median (IQR) - years	26 (16)	3	24 (31)	8	0.649
ACQ6 at enrolment, Median (IQR)	3.50 (1.90)	1	2.00 (1.80)	6	<0.001
SGRQ Score (Symptoms) at enrolment, Mean (SD)	73.58 (15.25)	5	57.98 (23.19)	17	<0.001
SGRQ Score (Activity) at enrolment, Mean (SD)	81.91 (17.14)	6	50.12 (25.29)	27	<0.001
SGRQ Score (Impacts) at enrolment, Mean (SD)	60.18 (16.93)	6	29.36 (17.56)	23	<0.001
SGRQ Score (Total) at enrolment, Mean (SD)	68.85 (13.86)	6	40.61 (18.26)	31	<0.001
Nijmegen score, Median (IQR)	37.0 (12.0)	5	13.0 (11.8)	25	<0.001
FeNO at enrolment, Median (IQR) (PPB)	68.6 (16.2)	9	26.7 (33.0)	48	0.015
Pre-BD FEV_1 predicted, Mean (SD) - %	72.7 (20.7)	25	74.4 (20.4)	100	0.804
Clinic (Post-BD) FEV_1 predicted, Mean (SD) - %	79.5 (20.8)	12	74.7 (22.3)	43	0.331
Pre-BD FVC predicted, Mean (SD) - %	83.1 (20.1)	25	90.9 (19.7)	100	0.245
Clinic (Post-BD) FVC predicted, Mean (SD) - %	88.3 (16.7)	12	91.3 (18.7)	43	0.481
Pre-BD MEF 25-75, median (IQR)	44.2 (69.8)	25	43.5 (44.2)	102	0.913
Clinic (Post-BD) MEF 25-75, median (IQR)	51.1 (72.2)	12	46.6 (45.9)	43	0.232
	**Percentage (frequency)**	**Missing**	**Percentage (frequency)**	**Missing**	**p-Value**
Sex, Male	30.6% (11)	0	47.7% (71)	0	0.064
Body Mass Index ≥30, Ever	62.9% (22)	1	38.5% (57)	1	0.009
Smoked, Ever	66.7% (24)	0	36.2% (54)	0	0.001
Adult-onset (≥18) asthma, Ever	42.4% (14)	3	44.0% (62)	8	0.872
Dysfunctional breathing, Ever	74.3% (26)	1	36.6% (53)	4	<0.001
Allergic Rhinitis, Ever	19.6% (11)	4	31.7% (45)	7	0.769
GORD, Ever	34.4% (11)	6	45.2% (66)	7	0.005
Other Psychological co-morbidity, Ever	17.9% (5)	8	4.7% (6)	17	0.013
Intermittent laryngeal obstruction, Ever	14.3% (4)	8	11.7% (16)	12	0.751

Nasal Polyposis, Ever	13.9% (5)	0	29.8% (42)	8	0.054
COPD, Ever	8.6% (3)	1	10.2% (15)	2	1.000
ABPA, Ever	2.9% (1)	3	10.9% (16)	2	0.203
Bronchiectasis, Ever	5.6% (2)	2	19.0% (28)	2	0.063
Urticaria/Angioedema, Ever	8.8% (3)	5	10.2% (15)	17	1.000
Sulphite sensitivity, Ever	0.00% (0)	3	7.5% (11)	13	0.220
Salicylate sensitivity, Ever	25.7% (9)	13	23.3% (34)	3	0.762
Eczema, Ever	11.4% (4)	1	25.9% (38)	2	0.069
Obstructive Sleep Apnoea, Ever	15.2% (5)	8	4.8% (7)	3	0.047
ICU admissions for asthma, Ever	33.3% (12)	0	27.5% (41)	0	0.488
Intubation for asthma, Ever	11.1% (4)	0	14.1% (21)	0	0.790
On maintenance OCS at enrolment, Yes	45.7% (16)	1	37.3% (53)	7	0.362
On Biologics at Enrolment, Yes	11.1% (4)	0	22.8% (34)	0	0.119

Severe psychological comorbidities= those with a clinical diagnosis of anxiety / depression + 'Severe' HADS-A /HADS-D. No psychological comorbidities = no anxiety, no depression + normal HADS-A and normal HADS-D.Continuous variables were expressed in median and interquartile range (IQR) or mean and standard deviation (SD) and analyzed with independent samples *t*-test or Mann-Whitney U tests. Categorical variables expressed as Percentage (frequency) and analyzed by Chi-squared tests. OCS: oral corticosteroids. ACQ6: asthma control questionnaire 6. COPD: Chronic Obstructive Pulmonary Disease. ABPA: Allergic Bronchopulmonary Aspergillosis. GORD: Gastro-eosophageal reflux disease. BD: Bronchodilator. FEV_1: forced expiratory volume in one second. FVC: forced vital capacity. MEF_{25-75}: Mean Mid Forced Expiratory flow rate. FENO: Fractional exhaled Nitric Oxide. ICU: Intensive care unit.
